# Hospitalization for ambulatory care sensitive conditions: What conditions make inter-country comparisons possible?

**DOI:** 10.1016/j.hpopen.2021.100030

**Published:** 2021-01-08

**Authors:** João Victor Muniz Rocha, Rui Santana, Juan E. Tello

**Affiliations:** a Escola Nacional de Saúde Pública, Comprehensive Health Research Centre, Universidade NOVA de Lisboa, Portugal; b WHO Regional Office for Europe, Denmark

**Keywords:** Ambulatory care sensitive conditions, Avoidable hospitalizations, Inter-country comparison, Scoping study

## Abstract

Hospitalizations for ambulatory care sensitive conditions have been extensively used in health services research to assess access, quality and performance of primary health care. Inter-country comparisons can assist policy-makers in pursuing better health outcomes by contrasting policy design, implementation and evaluation. The objective of this study is to identify the conceptual, methodological, contextual and policy dimensions and factors that need to be accounted for when comparing these types of hospitalizations across countries. A conceptual framework for inter-country comparisons was drawn based on a review of 18 studies with inter-country comparison of ambulatory care sensitive conditions hospitalizations. The dimensions include methodological choices; population’s demographic, epidemiologic and socio-economic profiles and features of the health services and system. Main factors include access and quality of primary health care, availability of health workforce and health facilities, health interventions and inequalities. The proposed framework can assist in designing studies and interpreting findings of inter-country comparisons of ambulatory care sensitive conditions hospitalizations, accelerating learning and progress towards universal health coverage.

## Highlights

• Inter-country comparison allows performance improvements based on evidence.
• Population-profile, health-system features and methodology are key dimensions in inter-country comparisons.
• Dimensions to account for depend on the objective and countries compared.
• Responsive primary health care is associated with lower avoidable hospitalization rates.

## 1 Introduction

It is commonly accepted that, for some health conditions, timely and adequate management, treatment and interventions delivered in the outpatient setting could potentially avoid the need for hospitalization. These conditions are known as *ambulatory care sensitive conditions* (ACSC) and they have been widely used as an indicator of access, quality and performance of primary health care and overall health services [1], [2], [3], [4]. The concept of analysing potentially avoidable hospitalizations started in the United States in the 1990s to evaluate access to health services [3]. It later expanded to other countries. Since then, there is a wide and growing body of literature on ACSC and, due to its usefulness, it has been endorsed by national stewards and international organizations as an indicator of performance [1], [2], [5], [6], [7].

There is evidence that features related to access, quality, integration and efficiency of services are positively associated with ambulatory care sensitive conditions hospitalizations (ACSC hospitalizations) [5]. Availability of health professionals and facilities, financial incentives, continuity of care, gatekeeping role of primary health care, monitoring of high-risk patients, among others are associated with ACSC hospitalizations [8], [9], [10], [11], [12], [13]. However, the severity of the disease and the patient underlying clinical conditions may influence the hospitalizations rates [14], [15], [16], [17]. ACSC hospitalizations are also positively associated with deprivation, unemployment, scarce education attainments, low level of income and rurality [8], [9], [18], [19].

ACSC hospitalizations may be unsafe and harmful for patients and their families, generate an additional burden for health professionals, create difficulties for health managers and policy-makers responsible for planning health services delivery and negatively impact the health system funding. Governments and international organizations are increasingly encouraging the development of primary health care and overall outpatient services as an alternative model to expensive hospital care. Comparative studies on ACSC hospitalizations across countries can indicate vantage points and achievable goals to improve services delivery, design interventions and reduce ACSC hospitalizations.

Comparisons across countries can accelerate health service and system improvements by providing valuable opportunities for contrasting experiences, stimulating inter-country learning and increasing policy options to act upon. The use of comparable indicators on the quality of health services can help countries assessing their situation and improving performance [20], [21]. Studies have shown that ACSC hospitalizations account for around 20% of total hospital admissions in England [22], Colombia, Argentina and Paraguay [23], around 13% in France [24] and 8% in Italy [25]. In Germany and Kazakhstan over 75% of hospitalizations for hypertension could have been avoided [26], [27]; in Portugal and Germany around 60% of hospitalizations for heart failure could have also been avoided [26], [28]. For diabetes, the percentage for avoidable hospitalizations has been found to range from 40% to 80% in Germany, Latvia and Moldova [26], [29], [30]. The use of ACSC hospitalizations to compare performance across countries may result in joint policy developments. One notable example is the Health Care Quality Indicators Project initiated in 2001. The Project measures and compares the quality of health care of different countries through a set of agreed indicators [31].

Despite these advantages, up to date, only few studies compare ACSC hospitalizations across countries and those available have different objectives and use different methodologies.

This study seeks to identify the conceptual, methodological, contextual and policy aspects that need to be accounted for when comparing ACSC hospitalizations across countries.

## 2 Methods

The starting point for this study was to review the published literature, research articles and grey literature, on ACSC hospitalizations inter-country comparisons. The review was conducted through searches in the electronic databases BioMed Central, PubMed and Web of Science using the terms “ACSCs”, “ACSC hospitalizations”, “avoidable hospitalizations”, “potentially avoidable hospitalizations” and “avoidable hospital conditions”. The search aimed to identify studies published in English from January 2000 to April 2019.

Those studies that compared numbers or rates of ACSC hospitalizations between two or across more countries were included regardless if the comparison was the main aim or part of a broader objective of the study. All studies were considered whether they analysed ACSC hospitalizations by single condition or aggregately. The references of the included studies were also reviewed to identify additional research. Abstracts without full articles and studies not published in English were excluded (one study in German and one in Portuguese). In total 390 studies were found. Out of this, 18 met the inclusion criteria and were reviewed.

The data analysis consisted of three steps. In a first step, the conceptual and methodological considerations of the included studies were examined. In a second step, the findings of the studies were clustered to identify common dimensions associated to ACSC hospitalizations. A third step consisted on analysing the policy implications of these studies and match them to their purposes and the countries involved. A conceptual framework for ACSC hospitalizations inter-country comparisons was derived.

This cross-sectional, scoping study aimed at outlining the factors that weigh inter-country comparisons. The study identifies limitations, and measures to overcome them, regarding the conceptualization, methodological aspects and contextual factors associated to ACSC hospitalizations.

## 3 Findings

Eighteen studies were identified and analysed. Table 1 shows the full-text articles and reports selected. Eight studies used only descriptive statistics to compare ACSC hospitalizations between countries [6], [22], [23], [24], [32], [33], [34], [35]. In four of these studies, comparison of ACSC hospitalizations was part of a broader objective of discussing health services performance [6], [22], [32], [35]. Nine studies employed additional statistical methods to explore possible associations with different variables [25], [36], [37], [38], [39], [40], [41], [42], [43]. Most of studies included only high-income countries [22], [25], [40], [42], [43], [32], [33], [34], [35], [36], [37], [38], [39]. Three studies targeted specific cities [36], [37], [41]. Three studies analysed only one specific health condition (diabetes) [38], [40], [41] and for two studies the conditions selected for analysis for each country were different [5], [24].

**Table 1:** Overview of studies included in the review.

Study	Cities/Countries	Objective	Methods	Conditions
Chau et al. (2013) [36]	HKG, London, New York	Compare and analyse ACSC hospitalizations as proxy for assessing access to primary care	Multiple logistic regression models to examine the possible association between ACSC hospitalizations and individual and neighbourhood-level variables	*
Degos and Rodwin (2011) [32]	FRA, USA	Highlight differences between care-centred and system-centred approaches	Review and discussion of evidence	*
Guanais, Gómez-Suárez and Pinzón (2012) [23]	ARG, COL, CRI, ECU, MEX, PRY	Compare and analyse ACSC hospitalizations and their economic effect	Descriptive statistics of ACSC hospitalizations and estimation of costs	**
Gusmano et al. (2007) [33]	England, FRA	Compare and analyse ACSC hospitalizations as proxy for assessing access to primary care	Comparison of age-standardized rates	*
Gusmano, Rodwin and Weisz (2006) [37]	Manhattan, Paris	Compare and analyse ACSC hospitalizations as proxy for assessing access to primary care	Multiple logistic regression models to examine the possible association between ACSC hospitalizations and individual and neighbourhood-level variables	*
Gusmano, Rodwin and Weisz (2014) [34]	England, DEU, FRA, USA	Compare and analyse ACSC hospitalizations as proxy for assessing access to primary care	Comparison of age-standardized rates	*
Kim and Cheng (2018) [38]	KOR, TWN	Compare and analyse hospitalizations for diabetes (an ACSC) as proxy for assessing quality of primary care	Multivariate, multi-level longitudinal models to examine the possible association between ACSC hospitalizations and individual and system-level variables	Diabetes
Kossarova, Blunt and Bardsley (2015) [22]	AUS, BEL, CAN, DEU, ESP, FRA, GBR, GRC, IRL, ITA, NLD, NZL, PRT, SWE, USA	Compare and analyse health care in the United Kingdom relative to other countries	Comparison of age-standardized rates	Asthma, COPD, diabetes
Kringos et al. (2013) [39]	AUS, BEL, CHE, CZE, DEU, DNK, England, ESP, FIN, GBR, ISL, IRL, ITA, LVA, MLT, NLD, NOR, POL, PRT, SVN, SWE	Compare and analyse ACSC hospitalizations as a proxy for assessing overall strength of primary care	Pearson correlation to examine the possible association between ACSC hospitalizations and variables on the strength of primary care	Asthma, COPD, diabetes
Loenen et al. (2016) [40]	AUS, AUT, BEL, CAN, CHE, CZE, DEU, DNK, England, ESP, FIN, HUN, ISL, IRL, ITA, LVA, NLD, NZL, NOR, POL, PRT, SVN, SWE	Compare and analyse hospitalizations for diabetes as proxy for comparing differences in the organization of primary care	Negative binomial analyses to examine the possible association between ACSC hospitalizations and variables on organizational characteristics of primary care	Diabetes
OECD (2017) [6]	AUS, AUT, BEL, CAN, CHE, CHL, COL, CRI, CZE, DEU, DNK, ESP, EST, FIN, FRA, GBR, HUN, ISL, IRL, ISR, ITA, JPN, KOR, LVA, LTU, LUX, MEX, NLD, NZL, NOR, POL, PRT, SVK, SVN, SWE, TUR, USA	Compare and analyse health outcomes and performance of health systems	Comparison of age and sex-standardized rates	Asthma COPD, diabetes, heart failure
Quan et al. (2017) [41]	HKG, JPN, Rural and peri-urban Beijing, SGP	Compare and analyse hospitalizations for diabetes (an ACSC) and their economic effect	Pearson correlation to examine the possible association between ACSC hospitalizations and variables on PHC use. Estimation of costs	Diabetes
Rosano et al. (2013) [25]	DEU, ITA	Compare and analyse ACSC hospitalizations as proxy for comparing differences in the health systems	Poisson regression models to examine the possible association between ACSC hospitalizations and contextual factors	***
Schiøtz et al. (2015) [42]	DNK, USA*	Compare and analyse ACSC hospitalizations as proxy for comparing differences in the organization of primary care	Logistic model to calculate the odds of rehospitalisation within 30 days after discharge for persons hospitalised with an ACSC	Angina, COPD, diabetes, heart failure, hypertension
Schneider et al. (2017) [35]	AUS, CAN, CHE, DEU, FRA, GBR, NLD, NZL, NOR, SWE, USA	Compare and analyse health care in the United States relative to other countries	Comparison of health system performance score	Asthma, Congestive heart failure, diabetes
Thygesen et al. (2015) [43]	DNK, England, ESP, PRT, SVN	Compare and analyse ACSC hospitalizations and variations	Exploratory multivariate regression models to examine the possible association between ACSC hospitalizations and contextual factors	Angina, asthma, COPD, dehydration, diabetes, heart failure
Weeks, Ventelou and Paraponaris (2016) [24]	AUS, BRA, CAN, CHE, DEU, DNK, ESP, FRA, GBR, IRL, ITA, PRT, SGP, SVN, USA	Compare and analyse ACSC hospitalizations in France with other countries	Comparison of age-standardized rates with results from previous studies	Varied
WHO (2016) [5]	DEU, KAZ, LVA, MDA, PRT	Review findings and the proposed conceptual framework for measuring ACSC hospitalizations	Stakeholder consultation. Estimation of rates of avoidability. Review and discussion of evidence	Varied

Abbreviations: ARG Argentina, AUS Australia, AUT Austria, BEL Belgium, BRA Brazil, CAN Canada, CHE Switzerland, CHL Chile, COL Colombia, CRI Costa Rica, CZE Czech Republic, DEU Germany, DNK Denmark, ECU Ecuador, ESP Spain, EST Estonia, FIN Finland, FRA France, GBR United Kingdom of Great Britain and Northern Ireland, GRC Greece, HKG Hong Kong, HUN Hungary, IRL Ireland, ISL Iceland, ISR Israel, ITA Italy, JPN Japan, KAZ Kazakhstan, KOR Republic of Korea, LTU Lithuania, LUX Luxembourg, LVA Latvia, MDA Republic of Moldova, MEX Mexico, MLT Malta, NLD Netherlands, NOR Norway, NZL New Zealand, POL Poland, PRT Portugal, PRY Paraguay, SGP Singapore, SVK Slovakia, SVN Slovenia, SWE Sweden, TUR Turkey, TWN Taiwan, USA United States of America.

Notes: Degos and Rodwin (2011) study is based on data analysis by Gusmano using data from 2004, which are also present in the study Gusmano, Rodwin and Weisz (2014). Schiøtz et al. (2015) study compared the Danish Health System with Kaiser Permanente, a not-for-profit managed care organization in the United States. Weeks, Ventelou and Paraponaris (2016) mimicked definitions of ACSCs used in previous studies. WHO (2016) asked health providers and other relevant stakeholders to select priority ACSCs.

* Asthma, bacterial pneumonia, cellulitis, congestive heart failure, diabetes, gangrene, hypokalaemia, immunisable conditions, malignant hypertension, perforated or bleeding ulcer, pyelonephritis, ruptured appendix [44].

** Anaemia, angina, asthma, cellulitis, congestive heart failure, COPD, dehydration, diabetes, ear, nose and throat infections, epilepsy, gastroenteritis, hypertension, immunisable conditions, nutritional deficiencies, pelvic inflammation, perforated or bleeding ulcer, pneumonia, pregnancy and birth related conditions, tuberculosis, urinary tract infection [45].

*** Angina, appendicitis with complications, asthma, congestive heart failure, diabetes, disorders of hydro-electrolyte metabolism, hypertension, nutritional deficiency, pelvic inflammation, perforated or bleeding ulcer, pneumonia, urinary tract infections [46].

Three studies apply ACSC hospitalizations to evaluate access to care [33], [36], [37]. These studies discuss how social, economic and health system barriers are associated to ACSC hospitalizations in countries with different health systems. Other eleven studies apply ACSC hospitalizations to evaluate performance of services, with access being one of its components [5], [6], [43], [25], [34], [35], [38], [39], [40], [41], [42]. These studies investigate how health services, particularly primary health care, improve health outcomes in terms of performance, quality, organization or effectiveness.

The definition of what is expected from primary health care and its gatekeeping role varies across studies. The research methodology and the conditions selected for analysis depend on the objective of the analysis, the scope of primary health care and the organization of the health services [8], [47], [48]. The effective gatekeeping role of primary health care in combination with higher or lower accessibility to inpatient care, lead to lower or higher rates of ACSC hospitalizations. Authors of five studies argue that the availability of hospitals lead to induced-demand for hospitalizations and emergency services [5], [23], [25], [34], [40]. Therefore, although ACSC hospitalizations are commonly associated with performance of primary health care, their analysis encompasses the whole health service delivery system [5], [25], [34], [36].

### 3.1 Findings emerging from the inter-country studies: conceptual and methodological

A first challenge inherent to the analysis of ACSC hospitalizations is to reach consensus on the concept of what is sensitive to ambulatory care, i.e., what conditions could have been avoided by timely and effective ambulatory care. Different lists of conditions have been developed (see seven of the reviewed studies [23], [25], [32], [33], [34], [36], [37]). The process to define the ACSC usually starts with a literature review followed by discussions and validation with clinicians and health managers in each country. Such process takes into account the organization of care, the disease prevalence, the socioeconomic and cultural characteristics of the population and the patient pathway in the context of each health system [47], [48]. Given that these factors vary among and within countries, there is no consensus on a definitive list of ACSC. For two studies, the conditions analysed for each country varied [5], [24].

The first list of ACSC hospitalizations was developed in the 1990s for analysing of hospital utilization in the United States of America [3]. In 2004, an adapted ACSC hospitalizations list was developed in Spain [4]. In 2009, Purdy *et al*. [47] combined the ACSC hospitalizations and obtained a common set of 36 diagnosis. The NHS England used a subset of 19 conditions, corresponding to 35% of all ACSC hospitalizations identified by Purdy *et al.* [47]. In 2013, Bardsley *et al*. [49] combined previous sets of conditions to develop a unique non-country specific ACSC hospitalizations list. Despite these attempts for moving towards a common agreed list of ACSC hospitalizations, countries have developed or adapted the lists to their national context.

Regarding the methodological aspects, important considerations arise for how hospital admissions information is obtained. The most common data source for studies on ACSC hospitalizations is administrative databases; all 18 studies analysed used official hospital discharge databases. The extraction of information was done directly from databases [24], [25], [36], [38], [42], [43] or retrieving it from other datasets [22], [39], [40]. These databases are available in most countries; thus, facilitating data collection. However, data are usually collected for reimbursing providers and, in some cases, refer only to publicly funded activities [36], [43]. The data collection process can be more difficult in countries with health systems based on private insurance [24]. Despite verifications [5], these administrative databases are susceptible to errors which lead to lower reliability and quality [24], [36], [38]. The inter-country comparisons of ACSC hospitalizations may also be undermined by differences in data availability.

Discrepancies in coding practices and disease classification systems may also affect the inter-country comparability. Some ACSC hospitalizations studies only take the secondary diagnosis into analysis [44], [45], [46]. For instance, the principal diagnosis recorded can be dehydration, a complication of diabetes, that may or may not be considered an avoidable condition, instead of the diabetes itself [6], [43]. How lower extremity amputation procedures are recorded can influence rate variations for diabetes across countries.

Different versions of the International Classification of Diseases (ICD) are used to record hospitalization data [36], [42], [43]. There are some inconsistencies between the structures of different versions of the ICD applied [50]. Six of the studies compare ACSC hospitalizations using codes from different ICD versions [5], [36], [38], [40], [41], [43]. In addition, data on the category level; i.e., the first three numeric or alphanumeric digits in the 9th and 10th version of the ICD, may not accurately describe the health condition of the patient [5]. Eight studies describe the ICD codes used to identify the ACSC hospitalizations using the first numeric or alphanumeric digits [5], [23], [24], [34], [38], [40], [41], [43]. However, subcategory digits, useful to provide specific information of the disease or condition, are not always available or require manual extraction [5]. This approach does not account for comorbidities, e.g., mental health conditions, immunosuppressed status or low physical mobility. In order to overcome some of these challenges, an approach to estimate the proportion of avoidable ACSC hospitalizations by national practitioners has been developed [5].

Some initiatives have been taken to mitigate these challenges. Many countries have been working on improving the quality and comparability of hospitalizations data, focusing on coding practices, dataset structure and data specification, many times linked to payment and reimbursement mechanisms [2], [51]. The Organisation for Economic Co-operation and Development (OECD), for example, supports the use of linked data using a unique patient identifier, as they are more robust and comparable across countries [51]. However, indicators based on linked data are often more complex to calculate. To deal with problems of misclassification in diagnosis coding or the use of different ICD versions, some studies have enlisted experts to review and validate codes [42], [43].

Six studies adopt rigorous exclusion criteria to allow for comparability. Out of these six, four studies exclude cases of inter-hospital transfer [6], [36], [41], [42], two studies exclude episodes in which patients died during the admission [6], [38], and three studies exclude cases of admissions with any diagnosis code or major diagnostic category (MCD) for pregnancy, childbirth and the puerperium (ICD-9: codes 630-677; ICD-10: codes O00-O99; MCD:14) [41], [42], [43]. However, not all countries have coding practices that would allow to apply similar exclusion criteria, for example, one study acknowledges that the exclusion of inter-hospital transfer could not be fully complied with by some countries [6]. Ten studies analyse specific age groups, mostly adults [6], [22], [25], [32], [33], [34], [36], [38], [42], [43]. Two studies use data of more than one year to avoid the effect of seasonal variations [36], [37], seven others to allow for longitudinal analysis [23], [24], [25], [38], [41], [42], [43]. The use of data from different years requires appropriate interpretation and comparison of trends to account for changes in the coding practices and other disrupting factors.

Most studies calculate age and gender-standardized rates using different standardization methods and different reference populations to account for differences in population structure. Other variables controlled for and included in statistical models were ethnicity and comorbidities [36], [37], [38], [41], however, these were only available at the individual-level. At the population level, studies also accounted for income, education level and rurality [36], [37], [38] as well as health services resources, such as density of physicians, primary health care centres and hospital beds [25], [36].

In order to account for positive association between the availability of hospital beds and the hospitalization rates three studies include hospitalizations for all causes [36], or for conditions which admissions were non-preventable, non-elective or referral-sensitive e.g., appendicitis, gastrointestinal obstruction, hip fractures, lower-extremity joint replacements and organ transplants [37], [42]. Three studies account for the prevalence of a disease to explain variations across countries [39], [40], [41]. All these adjustments, although beneficial for comparative analysis, are subject to the availability of data.

### 3.2 Findings emerging from the inter-country studies: contexts, systems and services

The concept of ACSC was introduced in the United States to analyse access and use of health services. All the reviewed studies find higher rates of ACSC hospitalizations in the United States of America compared to other countries [24], [32], [33], [34], [35], [36] or to the OECD mean [6]. Researchers associate these findings with barriers to accessing primary health care [35], [37]. Differences in ACSC hospitalizations odds are attributed to differences in ethnicity, benefits of being covered by health insurance and ecological factors measured through neighbourhoods by income level [36], [37].

ACSC hospitalization rates were used to assess performance of primary health care in six studies which argue that a responsive primary health care is associated with lower rates of ACSC hospitalizations [5], [25], [38], [39], [41], [42]. In these studies, the quality of health services delivery was assessed through different dimensions such as access, coordination, continuity of care and efficiency.

The availability of general practitioners or primary health care facilities is not always positively associated with lower ACSC hospitalizations. The number of general practitioners in Italy and Germany, for instance, was not found to be statistically significant in its association with the ACSC hospitalizations [25]. In London and New York, the density of primary health care physicians did not influence ACSC hospitalizations [36]. On the contrary, the absence of general practitioners, in interaction with other variables, contributed to higher ACSC hospitalizations in some European countries [5]. Despite the supply of health professionals is commonly used as a proxy for access to primary health care [10], [52], the studies reviewed provide inconclusive information on how the supply of health workforce and health facilities could be acted upon to reduce ACSC hospitalizations.

There are no unidirectional results regarding whether ACSC hospitalizations are induced by the availability of hospital beds. For four studies, higher ACSC hospitalizations are closely related to greater hospital bed supply [5], [23], [34], [40]. One study on diabetes find that hospital bed supply had a stronger effect on ACSC hospitalizations than continuity and coordination of care have [40]. For others four studies, the density of hospital beds is not related to ACSC hospitalizations [36], [37], [42], [43]. Three out of these four studies included hospitalizations for all causes, for marker conditions or referral-sensitive conditions as controls [36], [37], [42]. Marker conditions are those for which the probability of hospitalization is not influenced by ambulatory care, e.g. hospitalizations for appendicitis, gastrointestinal obstruction and hip fractures [37]. These three studies found great differences between ACSC hospitalizations and hospitalizations for other conditions across countries [36], [37], [42].

The analysis of the reviewed studies illustrate how countries can respond differently to similar health interventions. Since the ‘90s, Italy implemented policies to reduce the number of hospital beds discouraging inappropriate hospitalizations. More recently, Germany applied a similar initiative to reduce the costs of the hospital sector but did not led to significant results [25]. A more comprehensive and systematic approach to early detection of diseases, prevention programmes and self-management support explains differences between ACSC hospitalizations rates of Kaiser Permanente and Denmark [42]. Following an aggressive chronic care policy promoting coordination of care and health education introduced in 2001, the rates for hospitalizations for diabetes have decreased consistently between 2002 and 2013 in Taiwan. A similar policy in Korea was introduced in 2003 but implemented at slower pace; with limited administrative support and scarce financial resources from local governments, has started to show results only after 2011 [38].

The populations epidemiologic profiles explain some of the variations for ACSC hospitalizations. For example, across 35 countries, Mexico presented some of the lowest age and sex-standardized rates of hospital admissions for asthma, chronic obstructive pulmonary disease and congestive heart failure [6]. However, it presented more than twice the mean rate of OECD countries of hospitalization for diabetes. Diabetes is the leading cause of death and disability in Mexico [53]. In fact, Mexico was the only Latin American country for which diabetes represented the highest proportion of ACSC hospitalizations [23].

The selection of conditions influences the outcomes of the inter-country comparison study. For example, Mexico, Korea, Turkey and Ireland report the lowest or higher ACSC hospitalization rates according to the condition chosen [6]. Age and sex-standardized rates for diabetes varied 7-fold among OECD countries, 12-fold for congestive heart failure and 25-fold for chronic obstructive pulmonary disease [6]. One study finds differences in trends depending on the conditions studied. Trends in ACSC hospitalizations rates differ between acute and chronic conditions in Italy while in Germany, the increase is more drastic for chronic conditions [25]. Aggregating the hospitalization rates for different conditions into one single index can level-out the differences. However, inter-country comparisons by single conditions allow a deeper understanding of the factors associated with deviations.

Two studies argue that the prevalence of diseases and health status do not explain differences in ACSC hospitalization rates. Older people in Hong Kong had better health indicators than their peers in London, but London showed lower ACSC hospitalizations for this age group [36]. Differences in the prevalence of asthma and ischemic coronary conditions were only slightly higher in Denmark than in Portugal. However, the age and sex-standardized rates of ACSC hospitalizations in Denmark were nearly 3 times higher than in Portugal. Given that differences in the burden of diseases did not significantly affect rates, the authors believe that country specific factors influence health services delivery and explain the variations across countries [43].

There is a strong association between inequality and health status. Four of the reviewed inter-country studies found that people living in economically disadvantaged areas have higher probabilities of being hospitalized for ACSC [25], [36], [37], [38]. These findings, though, did not apply to four out of the five European countries analysed in another study [43]. Divergences in results regarding socioeconomic status can result from data availability and collection, conditions selected and method of analysis. Socioeconomic status as a proxy of people needs was mostly analysed at regional level.

Table 2 provides an overview of the key information analysed in the 18 studies and discussed in the sections above.

**Table 2:** Overview of the objectives, methods and findings of the 18 studies analysed.

Topic	Number of studies	Studies
*Objective of Study*		
Assess access to health care	3	[33], [36], [37]
Assess performance of health care	11	[5], [6], [43], [25], [34], [35], [38], [39], [40], [41], [42]
Compare ACSC hospitalizations	4	[22], [23], [24], [32]

*Setting*		
Cities	3	[36], [37], [41]
Two countries	5	[25], [32], [33], [38], [42]
Three or more countries	10	[5], [6], [23], [24], [32], [34], [35], [39], [40], [43]

Methods		
*ACSC hospitalizations analysis*		
Descriptive comparison of rates	8	[6], [22], [23], [24], [32], [33], [34], [35]
Statistical models	9	[25], [36], [37], [38], [39], [40], [41], [42], [43]

*Study design*		
Cross-sectional	11	[5], [6], [40], [22], [32], [33], [34], [35], [36], [37], [39]
Longitudinal	7	[23], [24], [25], [38], [41], [42], [43]

*Data source*		
Administrative database	18	[5], [6], [36], [37], [38], [39], [40], [41], [42], [43], [22], [23], [24], [25], [32], [33], [34], [35]

*Conditions included*		
Existing lists	7	[23], [25], [32], [33], [34], [36], [37]
One condition (diabetes)	3	[38], [40], [41]
Set of conditions	6	[6], [22], [35], [39], [42], [43]
Different conditions for each country	2	[5], [24]

*Diagnosis codes*		
ICD-9	7	[5], [25], [36], [38], [40], [41], [43]
ICD-10	11	[5], [23], [43], [24], [32], [34], [36], [38], [40], [41], [42]
Unclear ICD version	6	[6], [22], [33], [35], [37], [39]

*Diagnosis analysed*		
Principal only	11	[5], [6], [41], [23], [25], [32], [34], [36], [37], [38], [40]
Principal and secondary	3	[24], [42], [43]
Unclear	4	[22], [33], [35], [39]

*Exclusion criteria*		
Inter-hospital transfer	4	[6], [36], [41], [42]
Diagnosis codes or major diagnostic category for pregnancy, childbirth and the puerperium	3	[41], [42], [43]
Inpatient death	2	[6], [38]
Specific age groups	10	[6], [22], [25], [32], [33], [34], [36], [38], [42], [43]
Unclear/no exclusion criteria	4	[5], [24], [35], [39]

Findings		
*Responsive primary health care associated to lower rates of ACSC hospitalizations*		
Yes	6	[5], [25], [38], [39], [41], [42]
Inconclusive	1	[40]
*Availability of hospital beds associated to ACSC hospitalizations*		
Yes	4	[5], [23], [34], [40]
No	4	[36], [37], [42], [43]
*Association between Availability of GP and ACSC hospitalizations*		
Inverse	2	[5], [37]
Mixed results	2	[36], [41]
Non-significant results	2	[25], [38]
*Association between socioeconomic factors and ACSC hospitalizations*		
Yes	4	[25], [36], [37], [38]
Mixed results	1	[43]

Source: Elaborated by the authors.

### 3.3 Findings emerging from the inter-country studies: policy implications and knowledge translation

The comparison of health system performance across countries has been increasingly stimulated by the growing availability of data. Some of the reviewed studies analyse clinical practice variations among ACSC hospitalizations rates [6], [22], [23], [24], [33], [34], [35] while others explore possible associations with contextual factors, mostly through statistical methods [25], [36], [37], [38], [39], [40], [41], [42], [43]. The OECD Healthcare Quality Initiative uses admissions of ACSC to share and compare information on the performance of the health services across member countries [54].

The reviewed studies find high variability of ACSC hospitalizations across and within countries [6], [25], [43]. The analysis of rates, trends and inter-country variations allow to identify possible improvements in the quality of care in addition to efficiency gains. In a context of limited resources and increasing health expenditure, the possibility of decreasing spending by avoiding unnecessary or inappropriate hospitalizations is relevant to the national health agendas worldwide. One of the studies, for example, estimates avoidable hospitalizations and associated costs for countries without available data by using trends of other countries [23]. It could be argued that additional investment or more efficient allocation of existing resources towards strengthening primary health care would reduce ACSC hospitalizations and, consequently, decrease expenditure on the hospital care, which has notably higher individual costs [55], [56] and increased patient safety risks [57].

One of the analysed studies estimates that the 1.6 million ACSC hospitalizations in France had a total cost of 5 billion euros in 2010 [24]. Another study estimates that ACSC hospitalizations accounted for 2.4% of the public health expenditure of 26 countries in the region of Latin America and the Caribbean in 2009 [23]. Costs were estimated using unitary costs of the Brazilian public health system and adjusted by purchasing power parity in US dollars. Although the use of purchase power parity can be useful for inter-country comparisons [58], [59], spending associated to ACSC hospitalizations across countries remains challenging. Notably, in addition to the above-described methodological challenges, prices represent the values reimbursed to hospitals rather than real costs; differences in clinical practice weight in the procedures reimbursed. Other two studies acknowledge that reduced ACSC hospitalizations can lead to reduced hospital care expenditures; these studies however, did not estimate nor compare ACSC hospitalizations costs between countries [39], [41].

The factors associated to variations in performance have implications for health policies across countries. The relative success of specific health policies in Italy [25] and Taiwan [38], Kaiser Permanente [42], France and England [36], [37] cannot be adopted by policy-makers without taking into account the contextual factors of each health system [60]. There are many factors associated with ACSC hospitalizations which vary across countries and are sources of uncertainty. The transferability of policies and organizational characteristics is not, therefore, a straightforward process. For example, the reviewed studies are inconclusive regarding how the supply of general practitioners or of hospital beds affects ACSC hospitalizations [5], [25], [37], [38], [39], [40], [42].

Some studies compare countries with similar features. One study analyses Latin American countries at similar stages in the demographic and epidemiologic transition [23]. Another study compares South Korea and Taiwan, both countries have health systems based on social health insurance schemes and similar cultural heritages [38]. A study compares Italy and Germany, both European high-income countries [25]. The inter-country comparisons can also derive from aspects of divergence: Italy and Germany adopted different health systems, South Korea and Taiwan have differences in the organization and financing of primary health care as well as in how health policies were implemented. The inter-country comparison of ACSC hospitalizations can focus on performance of the health systems: three studies discuss financial barriers to access health care the United States of America in comparison to other countries [34], [36], [37]. Comparisons across health systems are useful since many challenges are common across countries: demographic and epidemiological changes, resource constraints and rising costs [60].

## 4 Discussion

Based on the above findings, three dimensions need to be accounted for in inter-country comparison of ACSC hospitalizations: methodological choices; population demographic and epidemiologic profiles and features of health services and systems. Table 3 provides an overview of the conceptual framework for inter-country comparisons of ACSC hospitalizations.

**Table 3:** Conceptual framework for ACSC hospitalizations inter-country comparison.

	Methods				Population	Health system
	ACSC	Data	Analysis	Study design		
Factors	Selection of condition and diagnostic codes	Representativeness	Unit of observation	Longitudinal vs cross-sectional analysis	Demographic structure	Gatekeeping role
	Inclusion/exclusion criteria	Reliability	Unit of analysis	Retrospective vs prospective	Epidemiological profile	Payment of providers
	Single vs multiple conditions	Co-morbidities and severity	Metrics		Socio-economic status	Availability of general practitioners
	Multiple hospitalizations and transfer	Coding practice			Geographic distribution	Availability of inpatient beds
						Public-private mix

### 4.1 Selecting the ACSC

The selection of the ACSC depends on the demographic and epidemiologic profile and the scope of primary health care services of a given country. There is significant variation of rates of ACSC hospitalizations across countries depending on which conditions are selected [24], [47], [61], [62]. For this reason, there is need to define consistent inclusion and exclusion criteria of cases e.g. make explicit if inter-hospital transfers, multiple hospitalizations (readmissions) or death of patients during the admission or which diagnosis codes will be included for a certain condition. Inter-hospital transfers and inpatient deaths may indicate that the hospitalization was ultimately not avoidable [6]. Some patients have multiple ACSC hospitalizations within a specific time frame. An option would be to count only one admission per patient if these are episode-based analysis. In any case, the methodological choice on how to account for these multiple hospitalizations related to a single patient would affect hospitalization-based rates [51]. The inter-country comparison of one single condition can be useful for deciding on a specific policy while the analysis of several conditions illustrates features in the assessment of the performance of health services.

### 4.2 Accounting for data configurations- data

The representativeness of data needs to be accounted for in ACSC hospitalizations inter-country comparisons. Data may be limited to public funded activities or be only available for a non-representative sample of the population. In some cases, hospitalizations compensate for inequities in access rather than clinical conditions e.g. social hospitalizations [63]. Not all ACSC hospitalizations are avoidable, in many cases due to comorbidities or the complexity and severity of cases. Variations in coding practices across countries and the use of different versions of the ICD may be also considered.

### 4.3 Choosing between one or more snapshot- analysis

Another methodological choice that affects inter-country comparison is the type of analysis. The choice between longitudinal and cross-sectional analysis will depend on the research questions. For instance: longitudinal comparisons can be useful to analyse the impact of health policies or changes in clinical practices while cross-sectional comparisons can be useful to analyse performance or to estimate efficiency gains. Inter-country comparisons can also be used for estimating ACSC hospitalization rates. The analysis of descriptive statistics can be suitable when comparing a wide variety of countries; more advances statistical models allows for more accurate inferences regarding the countries analysed. The methods of analysis will depend on the objectives and objects of comparison. Although no inter-country study that performed prospective analysis was found, it should be noted that adopting this type of analysis for inter-country comparison of ACSC hospitalizations needs to consider the complexity on data collection, limitation on external validity and necessary ethical considerations.

### 4.4 Observing units and analysing data- study design

Different units of observation and of analysis were found in the inter-country comparison of ACSC hospitalizations: by episode, patient, geographic area and provider. Different metrics are also used for analysis: ACSC hospitalizations can be measured in absolute number, rate, proportion of all hospitalizations or economic value.

### 4.5 Profiling the population

The profile of the population has significant impact in the analysis and need to be included in the inter-country comparison. These factors include the demographic structure of the population, its epidemiologic profile, socio-economic status and geographic distribution. The reviewed studies find high variability of ACSC hospitalizations across and within countries. The demographic and epidemiologic profiles of populations may explain some of these variations. Similarly, variations can be explained by the socioeconomic status of the population, since economically disadvantaged areas show higher rates of ACSC hospitalizations. To include individual information on socioeconomic characteristics of patients in inter-country comparisons is challenging since most administrative data is essentially used for reimbursement purposes. However, linkages among different databases may be possible.

### 4.6 Featuring services and system- health system

Features of health services and system to account for include the gatekeeping role, remuneration schemes, workforce distribution, public/private mix, coordination across providers and settings as well as the financing and availability of the hospital care; as found in the studies analysed. To account for features of health services and systems, including others outside the literature analysed, can be challenging, mostly due to difficulties in defining measurements and the unavailability of data.

### 4.7 Mitigating inter-country comparison limitations

There are possible steps that can mitigate limitations and improve comparability. The selection of avoidable hospitalizations can include codes for certain comorbidities, e.g. the methodology developed by the Agency for Healthcare Research and Quality [2]. Some countries use diagnosis-related groups to record hospitalizations and the level of severity can be accounted for. Experts can be consulted to estimate rates of avoidability [5], [64]. The use of single patient identifier on data can handle the existence of multiple counts of cases due to readmission. Comparing different populations may be possible by standardizing for age and sex and the controlling for prevalence rates and for socioeconomic status. The inclusion of hospitalizations for marker conditions in the analysis can help to account for the differences in overall hospitalization rates and practices among countries.

### 4.8 Limitations

The findings of this study have limitations. The inclusion criteria were narrowed to include full-text studies published in English. This resulted in the exclusion of two studies. One study, in German language, compares ACSC hospitalizations in Austria against other countries using OECD data but it did not discussed the method or interpret findings [65]. The second study, in Portuguese language, presents ACSC hospitalizations of two Brazilian cities and Spain to illustrate differences in the context [66]. These two excluded studies do not provide additional information to this study. The search-terms used might not have been comprehensive enough to retrieve all relevant studies. The scientific quality of the articles was not assessed. Despite these limitations, the approach adopted in this study allowed to examine methodological choices and to identify mitigating measures for the inter-country comparison of ACSC hospitalizations. The study findings align with our hypothesis and expectations.

## 5 Conclusions

Inter-country comparisons can assist policy-makers pursuing better health outcomes. The use of ACSC hospitalizations has the potential to signal suboptimal performance of services delivery. Inter-country comparison can help explain variations and explore policy options to improve practice based on evidence. This study proposes a framework to illustrate relevant dimensions and factors that need to be accounted for in inter-country comparisons of ACSC hospitalizations. The dimensions include methodological choices regarding selection, quality, treatment and analysis of information; populatiońs demographic, epidemiologic and socio-economic profiles and features of the health services and system. Factors to account for include access and quality of primary health care, availability of health workforce and health facilities, health policy interventions, and inequalities.

Despite this study advances methodological aspects and contextual policy implications, ACSC hospitalizations inter-country comparisons require caution. Most studies concur that the opportunities to reduce ACSC hospitalizations are mostly related to strengthening primary health care and promoting access, especially among more vulnerable populations but there is no agreement on how to target the root-cause of ACSC hospitalizations.

## Ethics approval and consent to participate

Not applicable.

## Consent for publication

Not applicable.

## Availability of data and material

Data sharing not applicable to this article as no datasets were generated or analysed during the current study.

## CRediT authorship contribution statement

**João Victor Muniz Rocha:** Conceptualization, Data curation, Funding acquisition, Investigation, Methodology, Writing - original draft. **Rui Santana:** Conceptualization, Validation, Writing - review & editing. **Juan E Tello:** Conceptualization, Funding acquisition, Methodology, Supervision, Validation, Writing - review & editing.

## Declaration of Competing Interest

The authors declare that they have no known competing financial interests or personal relationships that could have appeared to influence the work reported in this paper.
